# Supplementary material for: Prenatal Diagnosis Using Chromosomal Microarray Analysis in High-Risk Pregnancies
Source: J Clin Med. 2022 Jun 23;11(13):3624. doi: 10.3390/jcm11133624 (PMC9267905; doi:10.3390/jcm11133624)
Supplement: Supplementary file 1 [file jcm-11-03624-s001.zip › jcm-1747083-supplementary.pdf]

Supplementary Table S1. Overview of the cases with inherited benign, likely benign, or susceptibility copy number variants non-report after chromosome microarray (≧400 kb) in this study.

Case	No	CMA arr [hg19] results	Size (Mb)	Candidate OMIM genes	Diagnosis	Interpretation	Outcome
1	1	1q21.1(145,416,345-145,747,269)×3	0.33	605313	1q21.1 duplication	VOUS	Live born
2	1	1q21.3(152,200,830-152,822,826)×3	0.622	135940 / 616284	1q21.3 duplication	VOUS	Live born
3	1	2p25.3(343,867-840,548)×3	0.497	613220	2p25.3 duplication	LB	Live born
4	1	2q34(212,626,245-213,638,894)×3	1.013	600543	2q34 duplication	VOUS	Live born
5	1	3p26.3(61,891-1671,729)×1	0.944	607416 / 607220	3p26.3 deletion	LB	Live born
6	1	5p13.2p13.1(3,572,335-38,466,958)×3	0.895	600837	5p13.2p13.1 duplication	VOUS	Live born
7	1	6p22.2(26,017,272-26,642,819)×3	0.630	142220 / 613609	6p22.2 duplication	VOUS	Live born
8	1	7q21.12q21.2(88,004,021-91,808,838)×3	3.805	604001 / 617949	7q21.12q21.2 duplication	VOUS	Live born
9	1	8p22(12,939,619-13441,502)×3	0.512	604258	8p22 duplication	VOUS	Live born
10	1	8q24.11(118,244,105-118,883,898)×3	0.640	608177	Hereditary Multiple Osteochondromas	VOUS	Live born
11	1	11p13(31,693,465-33,036,716)×3	1.343	607102 / 607108 / 606985	11p13 WAGR syndrome duplication	VOUS	Live born
12	1	11p14.3p14.2(25,862,072-26,795,834)×3	0.934	610110	11p14.3p14.2 duplication	VOUS	Live born
		15q13.3(32,024,772-32,514,108)×3	0.489	612001	15q13.3 duplication	Benign	
13	1	11q14.114.2(84,895,612-85,661,235)×3	0.766	603583	11q14.1q14.2 duplication	VOUS	Live born
14	1	13q31.1(85,236,169-87,128,395)×3	1.892	609681	13q31.1 duplication	VOUS	Live born
15	3	15q11.2(22,765,628-23,217,514)×1	0.452	615656	15q11.2 deletion	VOUS	Live born
16	3	15q13.3 (31,819,442-32,444,070)×3	0.615	612001	15q13.3 duplication	Benign	Live born
17	1	16p12.2 (21,949,437-22,425,608)×1	0.476	136570	16p12.2 deletion	VOUS	Live born

18	1	21q21.1(20,641,135-21,265,393)×3	0.624	N/A	21q21.1 duplication	LB	Live born
19	1	Xp22.31(6,460,120-8,101,179)×2 (male)	1.641	308100	Ichthyosis, X-linked (XLI)	LB	Live born
					X-linked mental retardation (VCX3A)		
					Kallmann syndrome 1 (KAL1)		
20	1	Xp22.31(6,460,120-8,101,179)×3 (female)	1.641	308100	Ichthyosis, X-linked (XLI),	LB	Live born
		2q13(110,863,437-110,977,167)×2	0.114		Kallmann syndrome 1 (KAL1),		
21	1	Xp22.31(6,456,231-8,101,238)×3 (female)	1.645	308100	X-linked mental retardation,	LB	Live born
					X-linked mental retardation (VCX3A)		
					Ichthyosis, X-linked (XLI)		
22	3	Xp22.31(6,560,639-7,150,092)×3 (female)	0.589	308100	X-linked mental retardation,	LB	Live born
					Ichthyosis, X-linked (XLI)		
23	1	Xp22.31(7,304.945-7,809,343)×3 (female)	0.504	308100	Xp22.31 duplication	LB	Live born
		Xp22.31(8,449,343-8,923,515)×3	0.474	300836	Kallmann syndrome 1(ANOS1)	VOUS	
24	5	Yq11.223q11.23(25,091,073-26,271,921)×0	1.181	400041	Yq11.223q11.23 deletion	VOUS	Live born
25	1	Yq11.223q11.23(24,138,946-26,271,921)×2	2.133	400041	Yq11.223 duplication	VOUS	Live born
26	1	Yq11.223q11.23(24,117,947-26,271,921)×2	2.154	400042	Yq11.223q11.23 duplication	VOUS	Live born
		5p13.2(37,547,153-37,965,265)×3	0.418	600837	5p13.2 duplication	VOUS	
27	1	Yq11.223q11.23(25,857,405-28,103,771)×0	2.246	400042	Yq11.223q11.23 deletion	VOUS	Live born
		2p25.3(343,867-8,450,549)×3	0.497	613220	2p25.3 duplication	LB	
		3p26.3(119,190-1,087,902)×1	0.969	607416	3p26.3 deletion	VOUS	

CMA: chromosome microarray, MIN: Online Mendelian Inheritance in Man, VOUS: variants of uncertain significance, LB: likely benign
